# Liberal Prizes

**Published:** 1867-04

**Authors:** 


### Editorial.

### LIBERAL PRIZES.

The Mississippi Valley Association, at its late meeting, renewed its offers of a gold medal of the value of one hundred dollars for the most approved essay on Alveolar Abscess, and the same for the most approved essay on Diseased Antrum. And two of its members, Drs. W. H. Morgan and Samuel S. White, offer an award of two hundred dollars for the most approved essay on Preserving and Promoting the Health of Dentists.

The following are the regulations by which the competitors and the committees are to be governed:

1. Essays competing for the prizes must be in the hands of the committees as early as January 1st, 1868.

2. The committees have power to reject all essays presented if regarded as unworthy of the award competed for.

3. Rejected essays are to be promptly returned, or disposed of as directed by the writers.

4. Each essay is to be accompanied by a sealed envelope containing the author's name.

5. If an award is made, the committee is to seal up the approved essay till it is reported to the Association, when it and the accompanying envelope are to be opened.

6. The copyright shall belong to the Association.

7. The committee shall not permit any one else to inspect or see the copy of any essay till after making a report to the Association.

8. Competition for these prizes is not restricted to any profession or country.

Medical as well as Dental journals are requested to publish these propositions.

Essays competing for these prizes should be sent to Dr. George Watt, Cincinnati, Ohio, who is Chairman of both committees.

